# Automated biological target volume delineation for radiotherapy treatment planning using FDG-PET/CT

**DOI:** 10.1186/1748-717X-8-180

**Published:** 2013-07-12

**Authors:** Maximilian Niyazi, Sonja Landrock, Andreas Elsner, Farkhad Manapov, Marcus Hacker, Claus Belka, Ute Ganswindt

**Affiliations:** 1 Department of Radiation Oncology, University of Munich, Marchioninistr. 15, 81377, Munich, Germany; 2 Department of Nuclear Medicine, University of Munich, Marchioninistr. 15, 81377, Munich, Germany; 3 Hermes Medical Solutions, Skeppsbron 44, 11130, Stockholm, Sweden

**Keywords:** PET/CT, Planning study, BTV, Target delineation

## Abstract

**Background:**

This study compared manually delineated gross tumour volume (GTV) and automatically generated biological tumour volume (BTV) based on fluoro-deoxy-glucose (FDG) positron emission tomography (PET)/CT to assess the robustness of predefined PET algorithms for radiotherapy (RT) planning in routine clinical practice.

**Methods:**

RT-planning data from 20 consecutive patients (lung- (40%), oesophageal- (25%), gynaecological- (25%) and colorectal (10%) cancer) who had undergone FDG-PET/CT planning between 08/2010 and 09/2011 were retrospectively analysed, five of them underwent neoadjuvant chemotherapy before radiotherapy. In addition to manual GTV contouring, automated segmentation algorithms were applied–among these 38%, 42%, 47% and 50% SUV_max_ as well as the PERCIST total lesion glycolysis (TLG) algorithm. Different ratios were calculated to assess the overlap of GTV and BTV including the conformity index and the ratio GTV included within the BTV.

**Results:**

Median age of the patients was 66 years and median tumour SUV_max_ 9.2. Median size of the GTVs defined by the radiation oncologist was 43.7 ml. Median conformity indices were between 30.0–37.8%. The highest amount of BTV within GTV was seen with the 38% SUV_max_ algorithm (49.0%), the lowest with 50% SUV_max_ (36.0%). Best agreement was obtained for oesophageal cancer patients with a conformity index of 56.4% and BTV within GTV ratio of 71.1%.

**Conclusions:**

At present there is only low concordance between manually derived GTVs and automatically segmented FDG-PET/CT based BTVs indicating the need for further research in order to achieve higher volumetric conformity and therefore to get access to the full potential of FDG-PET/CT for optimization of radiotherapy planning.

## Introduction

Over the recent years [^18^F] FDG-PET imaging has become a valuable tool in oncology. Based on the higher sensitivity and specificity compared to conventional imaging modalities such as CT or MRI alone, PET is mainly employed for staging.

In addition, PET-CT may also be used as prognostic and/or predictive tool for radiotherapy [1] or combined radiochemotherapy [2]. In this regard, the outcome of head-and-neck cancer patients has been related to SUV changes in PET imaging [3]. Several tumour entities have been described in which data from PET-CT were predictive for pathological response, namely in rectal cancer [4,5], NSCLC [6] or oesophageal cancer [7,8]. PET/CT was even described to be complimentarily to a CT scan and being able to predict early recurrence in breast cancer [9]. Recent trials on Hodgkin’s disease are at least in part based on PET imaging and stratification is done according to PET results. Involved-node radiotherapy has been suggested as tool to further improve the therapeutic ratio by reducing radiation-induced toxicity [10].

Altogether, PET is a relevant tool for a personalization in radiotherapy providing patho-anatomical information, predictive data, also information on prognosis and may be used for adaptive planning, e. g. in head-and-neck cancer with anatomical changes during therapy, in future [11]. Due to its ability to provide complementary information (tumour extent and physiology) to MRI and CT, PET/CT has a potentially important place for radiotherapy planning [12,13]. In this regard, two aspects have to be distinguished: Firstly, PET imaging can be used for improved target volume delineation. Secondly, it may also be used for enhanced dose delivery to the target. For example, dose painting by contours (DPBC) consists of creating an additional PET-based target volume which will then be treated by a higher dose level [14-18]. In contrast, dose painting by numbers (DPBN) aims for a locally varying dose prescription according to the variation of the PET signal [19].

Many other tumour types are currently under investigation as PET provides additional information on the tumour extent, lymph node involvement and putative distant metastases [20,21]. Nevertheless, several problems have to be solved in future such as the inclusion of dynamic analyses or the correct thresholding procedures [22].

In order to compare manually delineated GTVs and automatically generated FDG-PET/CT based BTVs we retrospectively analysed a series of 20 consecutive patients who underwent planning PET/CT scanning at a dedicated PET/CT scanner in treatment position. Tumour types were restricted to lung cancer, oesophageal cancer and pelvic cancer mainly consisting of cervical cancer with macroscopic tumor only. For this purpose, volumetric and geometric relationships between BTVs and GTVs were assessed.

## Methods

### Patients

20 consecutive patients received a planning PET/CT and were categorized into three groups: lung cancer, oesophageal cancer and solid cancer of the pelvic region. Scans were performed between 08/2010 and 09/2011 at the Department of Nuclear Medicine of the University of Munich. Only patients with a macroscopic primary tumour and unified GTVs were included into the retrospective analysis. Five of these patients received neoadjuvant chemotherapy upfront to radiotherapy, among these four lung and one oesophageal cancer patient (median duration from the last cycle to the PET scan was 2 weeks).

### [^18^F]-FDG PET and PET/CT

Whole-body PET scans were acquired in three-dimensional (3D) mode extending from the proximal femora to the base of the skull or from the proximal femora including the skull (Biograph 64 TruePoint PET/CT, Siemens Medical Solutions or GE Discovery 690, GE Healthcare, Pollards Wood, United Kingdom).

After fasting for at least six hours, blood glucose levels were measured, a diuretic and an antispasmodic medication was administered intravenously, followed by bolus administration of [^18^F]-FDG (mean 228 MBq). Scans were performed with an emptied bladder. The emission sequence was initiated at approximately 60 min after intravenous injection of the [^18^F]-FDG. Attenuation scanning was obtained by CT. Two nuclear medicine physicians performed a consensus evaluation of the final PET/CT or PET images using a dedicated software package (Hermes Hybrid Viewer Version 1.4, Hermes Medical Solution, Stockholm, Sweden).

### Definition and quantitative analysis of tumour volumes

A consultant radiation oncologist without prior knowledge of the study delineated all GTVs. All the GTVs were mainly based on CT imaging and results of PET imaging were taken into account [23,24] without any systematic recommendation regarding thresholding or tracer amount (only the raw data as well as the report of the nuclear medicine expert were used as guidance for contouring within the axial CT slices); manually generated GTVs and their corresponding PTVs were used for treatment purpose in all patients included in this analysis.

A secondary automated lesion segmentation based on the given [^18^F]-FDG PET was performed using a dedicated software package (Hybrid Viewer, research version, Hermes Medical Solutions, Stockholm, Sweden) in two different ways: Based on maximal SUV (SUV_max_) thresholding and also according to the total lesion glycolysis (TLG) criteria described in PERCIST [25].

**Figure 1:**
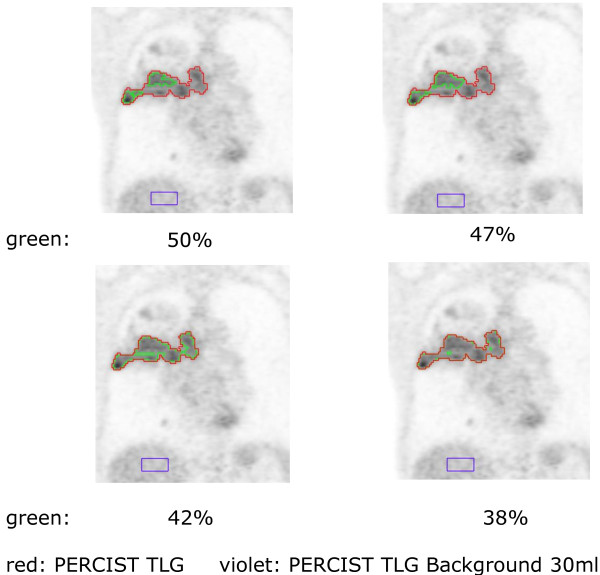
The principle of thresholding is shown for one patient with a NSCLC; red for the PERCIST TLG algorithm, green for different percentage threshold algorithms.

For the SUV_max_ thresholding criteria, separate VOIs were generated for 38%, 42%, 47% and 50% of SUV_max_ whereas these thresholds were chosen empirically based on literature, phantom studies and own experiences. The PERCIST TLG criteria were based on [^18^F]-FDG normal background activity determined from a 15 ml VOI in the right hepatic lobe. The outer boundary of the lesion is equal to 3 standard deviations (SD) above normal-liver mean SUV. Subsequently all five generated VOIs were back-projected onto the original CT images used for therapy planning and exported as DICOM RT structure sets for comparison with the manually contoured GTV [25] (an example is shown in Figure 1).

To assess geometric differences between BTV and GTV the conformity index was computed (expressed as percentage). It is defined as the ratio between intersection and conjunction of both volumes. Additionally the ratio intersection between GTV/BTV and GTV volumes was calculated to provide an estimate of accordance with the manually delineated gold standard.

### Statistical analysis

We performed all analyses using the Statistical Package for Social Sciences (SPSS, Ver. 18.0, SPSS Inc, Chicago, IL). For descriptive analyses of patients’ characteristics and volume sizes we used percentages and median scores. The GTV, and BTV delineation methods were compared using the Wilcoxon signed-rank test as numerical data were not normally distributed (paired tests); no corrections for multiple comparisons were performed. A two-tailed p-value of less or equal than 0.05 was considered to indicate statistical significance.

## Results

Altogether, among the examined 20 patients, eight had lung cancer (40%), five oesophageal (25%) and seven (35%) a cancer of the pelvic region (cervical/vulvar or rectal cancer). Thirteen patients were female (65%) and median age was 66 years (range, 34–93 years), mean age 66 ± 15 years. Median SUV_max_ was 9.2 (3.1–18.2), for an overview see Table 1.

**Table 1:** Patient characteristics

**Characteristic**	**Patients (N = 20)**
Sex	
• Male	7 (35%)
• Female	13 (65%)
Median Age [yr]	66 (34–93)
Tumour type	
• NSCLC	5 (25%)
• SCLC	3 (15%)
• Cervical cancer	4 (20%)
• Oesophageal cancer	5 (25%)
• Vulvar cancer	1 (5%)
• Rectal cancer	2 (10%)
Median SUV_max_	9.2 (3.1–18.2)

The PET/CT-based, manually contoured, GTV size was in median 43.7 ml. Concerning different thresholding algorithms, median volume of SUV (38%) was 29.2 ml, median volume of SUV (42%) 23.8 ml, of SUV (47%) 19.3 ml and of SUV (50%) 14.5 ml; these values are also shown for the three defined tumour type subgroups (see Table 2).

**Table 2:** Different thresholding volumes (median values), corresponding conformity indices and p-values of the comparison of these volumes to the GTV

	**Whole cohort**	**p-value (vs. GTV)**	**Median CI (GTV) [%]**	**Lung**	**p-value (vs. GTV)**	**Median CI (GTV) [%]**	**Oesophageal**	**p-value (vs. GTV)**	**Median CI (GTV) [%]**	**Pelvic**	**p-value (vs. GTV)**	**Median CI (GTV) [%]**
**Manual**												
**GTV [ml]**	43.7	--	--	81.1	--	--	34.2	--	--	70.4	--	--
**SUV(38%) [ml]**	29.2	0.04	32.9	30.9	0.33	24.0	15.2	0.04	51.9	28.7	0.24	29.8
**SUV(42%) [ml]**	23.8	0.02	30.0	25.4	0.21	25.5	13.8	0.04	47.6	26.0	0.24	27.2
**SUV(47%) [ml]**	19.3	<0.001	37.8	17.1	0.04	24.2	12.0	0.04	41.8	22.5	0.03	39.4
**SUV(50%) [ml]**	14.5	<0.001	33.3	14.3	0.03	20.5	11.4	0.04	38.7	22.0	0.02	34.5
**PERCIST TLG [ml]**	19.8	0.01	35.3	12.8	0.02	30.0	30.3	0.69	56.4	15.3	0.27	24.9

Interestingly, concerning the volumes there were significant differences between the SUV percentage thresholds/region growing algorithm and the manually defined GTV in all cancer types. Non-significant results were seen for oesophageal cancer when comparing the PERCIST TLG result with the GTV defined by the radiation oncologist, for lung cancer (SUV (38%), SUV (42%)) and pelvic cancer (SUV (38%), SUV (42%), PERCIST TLG) (see p-values in Table 2).

In a further step, conformity indices as well as the ratio of algorithm-defined volume within the GTV were evaluated for the whole patient cohort.

The best agreement was determined with a fixed SUV (38%) threshold: median 49% of this predefined volume was included within the radiation oncologist’s GTV. The remaining results are also shown in Table 2. Using the PERCIST TLG algorithm 43.7% were achieved.

Concerning conformity indices, the best agreement was determined with a fixed SUV(47%) threshold: median 37.8% overlapped with the GTV. Using the PERCIST TLG algorithm, the median conformity index was 35.3%. In a next step, the three subgroups defined by cancer site were analysed.

For lung cancer, the best agreement was determined by a fixed SUV (38%) threshold: median 35.4% of this automatically generated volume was included within the radiation oncologist’s GTV. Using the PERCIST TLG growing algorithm 30.2% of the respective BTV was included within the manually delineated GTV.

Contrarily, concerning conformity indices, the best agreement was determined with the PERCIST TLG algorithm: median 37.8% overlapped with the GTV.

For oesophageal cancer, the best agreement was found using the region-growing threshold: median 71.1% of this predefined volume was included within the radiation oncologist’s GTV. Concerning conformity indices, the PERCIST TLG again determined the best agreement: median 56.4% overlap with the GTV.

For pelvic cancer, the best agreement was determined by the SUV (38%) threshold: median 57.1% of this predefined volume was included within the radiation oncologist’s GTV. Concerning conformity indices, the best agreement was determined with the SUV (47%) algorithm: median 39.4% overlap with the GTV. For the remaining thresholds results were as follows: 29.8% (SUV (38%)), 27.2% (SUV (42%)), 34.5% (SUV (50%)) and 24.9% (PERCIST TLG).

## Discussion

Aim of this study was to examine in how far different PET segmentation algorithms have the potential to replace manually defined GTVs. Therefore, altogether 20 patients with cancer types of lung, esophagus or the pelvic region were considered, all of them bearing macroscopic tumours with no wide-spread disease.

Our data suggest that the general performance was rather limited with median conformity indices between 30.0–37.8%. The best match with the GTV was obtained for the subgroup of oesophageal cancer patients with a conformity index of 56.4% and BTV within GTV ratio of 71.1% whereas potential explanations are reduced movement artifacts and smaller well-defined lesions compared to lung or pelvic tumours. Especially the performance of percentage thresholding algorithms was poor–partly as they are derived from phantom studies (and empirically from retrospective series) where a SUV of 40–50% of the maximum was suggested to be appropriate for GTV contouring of sphere tumours with homogeneously distributed [^18^F]-FDG [26]. However, these thresholds having been derived under exactly reproducible scientific conditions had no dependence on SUV variations and background/noise changes seen in clinical situations.

Regarding these three tumour types, many data are available and studies are ongoing. However, most of them have not included a dedicated planning PET/CT but a separate scan which bears the disadvantage of co-registration errors.

PET/CT has early been shown to improve accuracy of target volume delineation for treatment optimization in NSCLC [27], but taking a 40% SUV_max_ threshold has been shown to be not appropriate for GTV definition [28]. Delineation studies exist showing that percentage volume changes from GTV [CT] to GTV [PET/CT] were lower for the nuclear medicine expert than for the radiation oncologists, suggesting a lower impact of PET/CT in target volume delineation for the nuclear medicine expert than for the radiation oncologists [29]; this observation is probably due to the radiation oncologist’s experience on the natural tumour spread and thus a tendency to extend the respective GTV.

Besides an improved target volume definition, PET has been shown to reduce radiation treatment volumes caused by the avoidance of PET negative mediastinal lymph nodes in NSCLC patients. Thus it reduces toxicity with the same radiation dose or enables radiation dose escalation with the same toxicity. PET also reduces interobserver variability [24,30] for delineating tumours and opens perspectives for more automated delineation parts in radiation treatment planning. Despite all inherent limitations present FDG-PET/CT scans are frequently used in routine clinical practice for NSCLC [31,32].

Concerning the pelvic region, with a constantly advancing body of evidence and increasing availability, applications of PET/CT in colorectal and cervical cancer are likely to emerge in therapy response assessment [33], radiotherapy planning, use of novel tracers and one-stop-shop imaging techniques such as iodinated contrast-enhanced PET/CT [34,35].

In a recent study comparing MRI and PET/CT in cervical cancer patients it could be shown that the mean difference between MRI and PET volumes was least with 30% SUV_max_ threshold which is similar to our results [36].

Last but not least, when combined PET/CT was used for radiotherapy treatment planning in oesophageal cancer, there have been alterations to the delineation of tumour volumes when compared to CT alone, with the potential to avoid geographical misses of tumour [37]. This indicates a clinical role for FDG/PET in radiotherapy planning for oesophageal cancer [38].

Related to the retrospective nature of our study with a relatively small heterogeneous patient cohort several key limitations have to be stated.

Firstly, due to subjective adaptation of the manually derived GTVs (where inter- and intra-observer variability were not taken into account), these may be larger than the real tumour extent. Secondly, since in 5/20 cases neoadjuvant chemotherapy was applied the given post-therapeutic tissue alterations may result in considerable problems for any auto-segmentation algorithm. Thirdly, co-registration was performed manually in a small number of cases leaving room for slightly imprecise correspondence of PET and CT information.

Furthermore, several minor problems have to be taken into account: Macroscopic tumour next to organs or structures with naturally higher glucose metabolism/uptake bears further problems for exact auto-segmentation as these structures have to be masked out manually. Additionally, distant secondary GTVs such as lymph node metastases may create problems related to their different FDG uptake pattern where a local background would be more suitable [39].

Thus, automated segmentation may indeed currently be used to complement the delineation process. But at present, none of the tested algorithms is able to fully replace the process of manual target volume definition and critical editing of any given PET signal.

From a radiation oncologist’s point of view the most immanent need would be the development of robust algorithms for the reliable definition of macroscopic tumour extent or adequate processing constraints to generate CTV [39,40] or PTV [41]. The most promising algorithms are background- and metabolically corrected algorithms such as the PERCIST TLG algorithm.

In the meantime, the use of PET for planning purposes requires a very close interaction of radiation oncologists, nuclear medicine experts and diagnostic radiologists for individual treatment planning. In this regard, several logistic issues have to be solved adequately: Who sets the contour at which time point, and how can all the available diagnostic information properly be integrated during the planning process?

## Conclusions

All in all, there is only low concordance between manually derived GTVs and automatically segmented FDG-PET/CT based BTVs at present indicating the need for further research in order to achieve higher volumetric conformity. Nevertheless, our data suggest that FDG-PET/CT may be regarded as an important tool for optimization of radiotherapy planning.

## Competing interests

Andreas Elsner due to employment. The remaining authors declare that they have no competing interests.

## Authors’ contributions

UG, MH, AE, SL, FM, CB & MN planned, coordinated and conducted the study. MN analysed the treatment planning data, fused imaging data set, performed associated statistics and wrote the manuscript. SL, AE & MH performed PET imaging, provided the standard for PET-contouring, the fused data sets and contributed parts to the manuscript. All authors read, critically revised and approved the final manuscript.
